# Carbamazepine and Multiple Myeloma: Possible Interaction

**DOI:** 10.4274/tjh.2012.0153

**Published:** 2013-03-05

**Authors:** Meral Günaldı, Semra Paydaş, Çiğdem Usul Afşar, Berna Bozkurt Duman, Vehbi Erçolak, Veysel Haksöyler

**Affiliations:** 1 Çukurova University Faculty of Medicine, Department of Medical Oncology, Adana, Turkey; 2 Çukurova University Faculty of Medicine, Department of Internal Medicine, Adana, Turkey

**Keywords:** Multiple myeloma, Carbamazepine, Epilepsy

**To the Editor**

Epilepsy is the most common chronic neurological disease and patients are treated by various classes of antiepileptic drugs [1]. In addition to their acute side effects, there are long-term adverse effects of antiepileptic drugs [2]. Hemopoietic neoplasias such as lymphoma, multiple myeloma, and some solid cancers including lung, liver, pancreas, and gastrointestinal cancers are the malignant disorders most frequently discussed in relation to use of antiepileptic drugs [3,4]. The carcinogenic effect of carbamazepine is very limited. However, there are some case reports and series containing a limited number of cases of hypogammaglobulinemia, monoclonal gammopathy of undetermined significance (MGUS), and multiple myeloma [3,4,5,6,7,8,9,10,11]. We present here a 54-year-old woman with multiple myeloma with long exposure to carbamazepine. Vertebral fracture was detected and vertebroplasty was done urgently. There was no evidence of neurologic deficit. She had a history of 400 mg carbamazepine usage for more than 20 years due to idiopathic epilepsy. Laboratory examination showed total protein/albumin of 8.85/3.41 g/dL and calcium of 10.77 mg/dL (8.4-9.2); she had macrocytic anemia. Bence-Jones protein in the urine was not demonstrated. Protein electrophoresis showed an M-peak (Figure 1) with an elevated serum level of immunoglobulin G (IgG) of 2740 mg/dL. Immunoelectrophoresis revealed M-protein composed with a kappa chain. Bone marrow aspiration and biopsy showed 90% plasma cell infiltration and CD38 and kappa were positive. Cytogenetic analysis of bone marrow showed 17 p deletion (+), 13 q deletion (+), t (11;14), t (4;14). Histopathological examination of a vertebroplasty specimen revealed plasma infiltration. Carbamazepine usage was stopped and treatment was continued with valproic acid. A regimen containing bortezomib (1.3 g/m^2^), dexamethasone (40 mg), and zoledronic acid was prescribed. After 4 courses of chemotherapy, blood and bone marrow exams were normal. Stem cell transplantation was planned for consolidation. Carbamazepine-related multiple myeloma has been reported in a few case reports. It has been suggested that carbamazepine may cause the IgG type of M-protein multiple myeloma, MGUS, and hypogammaglobulinemia [5,6,9,11,12]. Our patient was younger than is usual for multiple myeloma. We can speculate that the exposure to an extrinsic factor may be associated with younger age in this case. The relationships among the duration of use of antiepileptic drugs, cumulative dose of antiepileptic drugs, and development of multiple myeloma are not known completely. In our case, multiple myeloma developed after 20 years of carbamazepine use. In conclusion, carbamazepine may be a potential drug in the development of multiple myeloma. For this reason, periodical evaluation of serum levels of immunoglobulin is necessary in patients receiving carbamazepine.

**Conflict of Interest Statement**

The authors of this paper have no conflicts of interest, including specific financial interests, relationships, and/ or affiliations relevant to the subject matter or materials included.

## Figures and Tables

**Figure 1:**
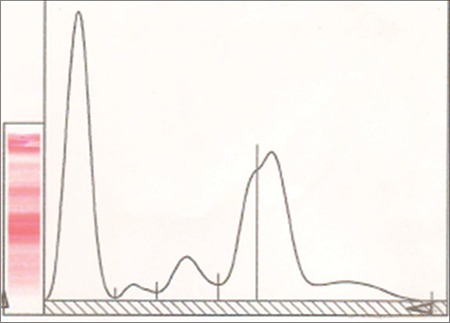
Abnormal serum protein electrophoresis pattern in a patient with multiple myeloma. Note the large spike in the early gamma region.
